# Cow's Milk and Rickets

**Published:** 1908-05-16

**Authors:** G. Arbour Stephens

**Affiliations:** 61 Walter Road, Swansea


### COW'S MILK AND RICKETS.

Sir,—The letter (written as a comment on my letter in your issue of the 2nd inst.) of your correspondent "J." is evidently meant for a joke. It makes one feel inclined to ask "Who is J., what is J., and even where is J.?" Wright apparently, according to this gentleman, is the panacea for almost everything, though, notwithstanding this panacea, rickets seem very prevalent. That the milk of to-day is not what it ought to be is evidenced by the numerous advertisements of different kinds of baby-foods that one sees in the daily and weekly, to say nothing of the medical, press.

Wright's propagandist states that the proportion of salts in cow's milk to that of human milk is as 7 to 3, and yet further on he points out the necessity of diluting the milk. The usual dilution, as "J." possibly knows, is about 3 or 4 of water to 1 of milk, which reduces the proportion of salts to 1½ to 3, a reduction that is quite sufficient to account for rickets. If "J." is right the wonder is that our blood can indulge in any circulation at all, after having been fed on "medicine" for such a number of years. Rather it ought to be one huge clot!

A correspondent with such opsomaniac tendencies as "J." seems to possess towards calcium is very likely to overlook the fact that in gestation there are many progressive protoplasmic changes, which may have as much to do with the disagreeing qualities of the milk as have the calcium salts. I venture to think that the facts to which I have drawn attention are quite sufficient to put an end to the ridiculous fiction of getting milk for the baby from one cow, for in the simultaneously increasing demands of embryonic calf and baby the latter is sure to suffer.

Yours truly,
G. Arbour Stephens.

61 Walter Road, Swansea, May 10, 1908.

